# The Effect of Granulometry of Carbonaceous Materials and Application Rates on the Availability of Soil-Bound Dichlorodiphenyltrichloroethane (DDT) and Its Metabolites

**DOI:** 10.3390/jox14010016

**Published:** 2024-02-11

**Authors:** Farida Amutova, Ronagul Turganova, Gaukhar Konuspayeva, Sarra Gaspard, Aigerim Mamirova, Florentin Michaux, Pamela Hartmeyer, Claire Soligot, Leyla Djansugurova, Stefan Jurjanz, Matthieu Delannoy

**Affiliations:** 1 Université de Lorraine, INRAE, URAFPA, F-54000 Nancy, France; amutovafb@gmail.com (F.A.); ronagul.turganova@univ-lorraine.fr (R.T.); pamela.hartmeyer@univ-lorraine.fr (P.H.); claire.soligot@univ-lorraine.fr (C.S.); stefan.jurjanz@univ-lorraine.fr (S.J.); 2 Al-Farabi Kazakh National University, Faculty of Biology and Biotechnology, Almaty 050040, Kazakhstan; konuspayevags@hotmail.fr (G.K.); aigerim.mamirova@mail.com (A.M.); 3 Antigen LLP, Scientific and Production Enterprise, Almaty 040905, Kazakhstan; 4 Laboratoire COVACHIM-M2E, EA 3592, Université des Antilles, F-97110 Pointe-à-Pitre, Guadeloupe, France; sarra.gaspard@univ-antilles.fr; 5 Université de Lorraine, LIBio, F-54000 Nancy, France; florentin.michaux@univ-lorraine.fr; 6 Institute of Genetics and Physiology, Almaty 050060, Kazakhstan; leylad@mail.ru

**Keywords:** environmental availability, sequestration, biochar, activated carbon, amendment rate

## Abstract

Biochars (BCs) and activated carbons (ACs) are well-known carbon-rich materials that are being increasingly studied in environmental sciences for water treatment applications to remediate pollutant sequestration in soil. This study aimed to assess the impact of Sargasso BC particle size and amendment rate on the environmental availability of DDT and DDT metabolites in two distinct Kazakh soils. These two soils were collected in the vicinity of storehouse facilities in Kyzylkairat and Beskainar that store banned pesticides. They presented very distinct concentration levels of DDT and DDT metabolites. Three different types of carbonaceous matrices were tested: Sargasso BC and two commercial ACs (ORBO^TM^ and DARCO^©^). For the granulometry effect, Sargasso BC was ground, and two particle sizes were tested (<150 µm, >150 µm) and compared to an unground material. Four distinct application rates were tested (0.25, 0.5, 1, and 2% (*w*/*w*)). After a three-month maturation period, environmental availability was assessed using an ISO/DIS 16751, part B-modified methodology. Interestingly, the best reductions in DDT environmental availability were obtained with the finest particle size (both ACs and Sargasso BC < 150 µm). More specifically, the effectiveness of the strategy seemed to depend on many factors. Firstly, a clear soil effect was demonstrated, suggesting that the more contaminated the soil, the more efficient this strategy may be. Secondly, the results showed that an increase in the amendment rate improves the immobilization of DDT and DDT metabolites. The sequestration material demonstrated different efficiency values (up to 58 ± 4% for Sargasso BC < 150 µm and 85 ± 4% for DARCO at a 2% application rate). Finally, a clear molecule effect was displayed, demonstrating the following immobilization order: p,p’-DDE > p,p’-DDD > p,p’-DDT > o,p’-DDT.

## 1. Introduction

Organochlorine pesticides (OCPs) were extensively used worldwide during the 20th century as an effective means of preserving crop cultures from deterioration due to insects, and they were included in sanitary policies to limit the spread of disease carriers for humans and animals [1,2,3]. Although their use highly improved aspects of both food production and disease control, the downsides of this methodology were revealed well after when environmental and toxicological considerations began to arise in the late 1960s [4]. The pesticide 1,1,1-trichloro-2,2-bis(p-chlorophenyl)ethane (DDT) and its metabolites are paragons of this flow of history. Having been found to have insecticidal and acaricidal properties in the late 1930s, DDT was the most used pesticide after World War II (400,000 tons used annually) [2]. Toxicological studies from the 1960s pointed out that the use of DDT can induce tremendous neurological, endocrinal, and oncological effects due to its high ecotoxicity [5]. Due to its persistency in soil (caused by its high partitioning onto organic matter), such contamination could last decades, with soil acting as a reservoir of this pollution. In spite of this high affinity to soil, such molecules transfer highly to biota and are bioaccumulated thereafter [6,7,8].

In Kazakhstan, such pesticides were used until 1990, even though a formal ban outlawing their use in agricultural practice was established in the 1970s. The remaining banned pesticides were collected and stored in several storage facilities throughout the country until further processing. Due to degrading conditions, confinement at these facilities has begun to fail, and the spread of DDT has arisen in the past decades. The villages of Kyzylkairat and Beskainar in southeast Kazakhstan are two examples of places where the past use of DDT and possible DDT leakage from these storage facilities are causing environmental problems. A major problem of such contamination is the heterogeneity of its extent and the concentrations of DDT found onsite even at relatively small-scale sites [9]. Nevertheless, even on moderately contaminated sites, the agronomic production services offered by the soil are put at risk, as the contamination of soil may transfer to the resulting food products [10,11,12,13,14,15]. Thus, without extensive concentration data, risk management regarding such contamination results in the avoidance of agronomic production in these contaminated areas, limiting food resources needed by locals.

Another way of limiting the transfer of DDT may be to remediate potentially contaminated areas in order to rehabilitate soil production services. Despite having tangible proof of natural degradation and the possible means through which the chemical or biological degradation of such molecules occurs, remediation appears expensive, presents yields with high variability, and is relatively time consuming [16,17,18]. In this context, the soil’s production function may be preserved by acting only on the transfer of such molecules to agronomic resources (cultivated vegetables, farm animals). Sequestration strategies involving soil treatment by using carbon-rich materials such as biochars (BCs) or activated carbons (ACs) have shown interesting efficiency and represent a promising way [19] of notably reducing transfer to mammals and ensuring the safety of their food products [6,20]. Due to their porous structures, which allow for the adsorption of organic contaminants on their large surfaces, these materials are known to efficiently adsorb organic contaminants [21]. One strategy has proven effective in limiting transfer to different agronomic biota such as plants and farm animals and in acting against different organic pollutants with high lipophilicity, such as PCBs, dioxins, and furans, as well as the OCP chlordecone (CLD) [10].

Recent studies have demonstrated important effects precursor sources (i.e., feedstock) have on the physicochemical properties of BCs and ACs, and therefore on their adsorption or sequestration efficiency [22,23,24]. However, concerning lipophilic organic pollutants, a clear difference in efficiency between BCs and ACs can be found in the literature. Indeed, if both porous materials can be produced from different raw materials such as plants or animal by-products [21], the activation process appears to be a key parameter in the adsorption of soil POPs. Such a process is known to enhance the porous surface as it limits residues on the surface of the pyrolyzed material [21]. However, due to the process needed, such materials are known to be expensive and may involve chemicals whose use raises environmental concerns. Recently, Ranguin et al. [25] discovered that *Sargassum* spp., a brown algae endemic to the Caribbean region that becomes toxic when decomposed, representing an available biomass that is not valorized, can be used as a precursor material for BCs. This preliminary study illustrated that BCs obtained from massive and easily available *Sargassum* spp., following pyrolysis at 700 °C for 3 h, exhibit notable characteristics such as significant porosity and a substantial specific surface area. These properties make them a promising candidate for adsorbing CLD, resulting in a reduction of more than 80% in CLD’s environmental availability in contaminated Nitisol [25]. These encouraging findings warrant further exploration to determine whether this efficiency could also prove beneficial for addressing other prevalent contaminants such as DDT and its metabolites, which are present not only in Kazakhstan but also in the French West Indies.

The aims of this study were (i) to characterize the effect of BC particle size and soil amendment rate on the environmental availability of DDT and DDT metabolites to select the most efficient strategy to reduce their availability and (ii) to compare the selected strategy with two commercial ACs known to reduce the availability of POPs.

## 2. Material and Methods 

In the current investigation, three diverse porous adsorbents were evaluated: one BC (Sargasso biochar) and two commercial ACs (DARCO and ORBO). The experimental design consisted of two phases: the initial phase focused on selecting the optimal Sargasso BC granulometry and amendment rate for effectively sequestering DDT and its metabolites, while the subsequent phase involved comparing this approach to AC-based sequestration methods. Two distinct soils contaminated with DDT were utilized in these experiments, each presenting varying contamination levels. The first soil sample originated from the village of Kyzylkairat (referred to as K) and exhibited high contamination, while the second soil sample collected from Beskainar (referred to as B) had a lower level of contamination.

### 2.1. Preparation of Sargasso Biochar 

Three distinct carbonaceous matrices were derived from Sargassum spp. The production process of the original Sargasso BC (referred to as Sarg) is comprehensively detailed in Ranguin et al. [25]. From this initial material, two additional BCs were synthesized. The Sarg BC underwent a 2-min grinding process using an analytical grinder (IKA Tube-Mill 100, Staufen, Germany) and was subsequently sieved using a 150 µm apparatus. Two particle size fractions were distinguished: the fraction sieved up to 150 µm (referred to as Sarg<) and the residue from the sieving process containing larger particles (referred to as Sarg>). 

### 2.2. Characterization of the Sequestering Matrices Particle Size 

In addition to the three distinct samples of BCs (Sarg, Sarg<, and Sarg>), two commercial ACs (DARCO and ORBO) were included. To evaluate the impact of particle size on the resulting bioavailability, the particle size distribution was characterized using a laser diffraction apparatus (Mastersizer 3000, Malvern Panalytical, Palaiseau, France). Laser diffraction analysis was conducted on three sample groups based on treatment: Group 1—untreated ACs, Group 2—BC Sarg with and without grinding, and Group 3—BC ground and sieved (Sarg< and Sarg>).

Briefly, this analysis employed two lasers with distinct wavelengths: a He-NE red light at 632.8 nm and a blue LED light at 470 nm. Approximately 20 mg of each granulometric fraction of both BCs and activated carbon (as described previously) was suspended in 10 mL of ultrapure water. The subsample was introduced into the Mastersizer 3000 dispersion unit with a maximum stirring speed of 3500 rpm, and 100% sonication power was applied throughout each measurement event (approximately 2 min). Analyses were conducted 15 times for each sample. Following dispersion, the sample circulated in the measurement cell where diffraction was measured. Very low variability (CV less than 10%) was observed. The particle size distribution of ACs and BC samples was determined using the device acquisition software (Mastersizer 3000, version 3.81, Malvern Panalytical, France), averaging the results of 15 consecutive analyses. 

### 2.3. Characterization of the Sequestering Matrices Porous Surface 

The specific surface areas of ACs were extracted from previously published data [25]. The specific surface areas (SSA) and porosity of the investigated BCs were determined at the LIEC laboratory (LIEC, Vandœuvre-les-Nancy, France) and are presented in Table 1. These measurements were conducted using volumetric nitrogen adsorption–desorption at 77 K. Nitrogen adsorption–desorption isotherms were recorded on a Belsorp max II (MicrotracBEL Corp., Osaka, Japan) equipped with a turbomolecular pumping unit that ensures a residual vacuum of 10−5 Pa. The device features four ports, including three for samples and one reference port. Each sample port is equipped with three pressure sensors covering different ranges: 133 kPa (1000 Torr), 1.33 kPa (10 Torr), and 0.0133 kPa (0.1 Torr).

After subjecting the samples to outgassing at 30 °C for 12 h under a residual vacuum of 10−5 Pa, nitrogen adsorption–desorption isotherms were conducted. These isotherms depict the volume of nitrogen adsorbed at 77 K against the relative pressure (P/P0), where P represents the equilibrium pressure of the adsorbing gas and P0 is the vapor saturation pressure. The measurements were performed using a step-by-step method within the interval of relative pressures, P/P0, up to 0.98. All experiments utilized ultrapure nitrogen (>99.9995%, supplied by Air Liquide). The Brunauer–Emmet–Teller (BET) method, employing a 16.3 Å cross-sectional area of nitrogen molecules [26], was employed to estimate the specific surface area (SSA). The De Boer method, also known as the t-plot [27], was applied to determine the microporous volume and external surface area. Micropore filling occurs at low and very low relative pressure values, encompassing the domain of monolayer adsorption on the external surface. To distinguish adsorption onto the external surface from adsorption into micropores (pore size < Å), the experimental isotherm was compared to a reference curve obtained for a non-porous solid with chemical features and energetic constants as closely matched as possible to the studied matrix. 

### 2.4. Soil Sampling and Amendment of Soil 

Soil samples were collected from two villages in the Almaty region (southeast Kazakhstan): Kyzylkairat (GPS 43°17′58″ N; 77°11′39″ E) and Beskainar (GPS 43°19′27″ N; 77°06′20″ E), in the vicinity of local storehouses for obsolete and banned pesticides. These soils are classified as kastanozems according to the World Reference Base for Soil Resources Classification [28].

The collected soil samples were air-dried until mass stabilization was achieved and then sieved through a 250 µm sieve. Subsequently, the soils were amended with one of five sequestering materials at rates of 0.25, 0.5, 1, or 2% (*w*/*w*). After hand mixing each sample for 10 min, water was added to achieve a humidity level of up to 15%, and the mixture was allowed to age for 3 months at ambient temperature (20 ± 2 °C). Each amendment was replicated four times (*n* = 4). For each soil, eight subsamples were left non-amended as controls (C) (*n* = 8). Concentrations of DDT and DDT metabolites were assessed at the LABOCEA laboratory for both soils located in Quimper, France, and the values are presented in Table 2.

### 2.5. Assessment of DDT and DDT Metabolites’ Environmental Availability

Environmental availability assays were conducted on each soil sample following a protocol adapted from XP ISO/TS 16 751 Part B, as previously detailed elsewhere [25]. In essence, 600 ± 25 mg of Tenax^®^ (60–80 mesh, Sigma-Aldrich, Saint-Louis, MA, USA) and 28 mL of ultrapure water were introduced to soil samples along with 13C labeled o,p’-DDT (Cambridge Isotope Laboratories, Tewksbury, MA, USA). Following a 20-h agitation period, Tenax^®^ was recovered, and DDT and its metabolites were subsequently extracted three times with acetone (LV-GC, Biosolve, Lyon, France) using an ultrasonic bath. All extracts were combined and concentrated before undergoing GC-MS/MS analysis. 

### 2.6. GC-MS/MS Analysis 

Analysis of DDT and its metabolites was conducted using a gas chromatograph coupled to a triple quadrupole mass spectrometer (TSQ 9000 GC-MS/MS ThermoFisher Scientific, Waltham, MA, USA), equipped with a DB-5MS (15 m, 0.25 mm, 0.10 µm) silica capillary column (ThermoFisher Scientific). Splitless injection was carried out on a PTV injector set at a constant temperature of 280 °C. Helium was used as a carrier gas with a constant flow rate of 1.2 mL/min, and argon served as a collision gas. The analysis proceeded as follows: starting at 100 °C with a gradual heating to 125 °C at a rate of 10 °C/min, followed by a gradual increase to 140 °C at a rate of 1 °C/min, holding at 140 °C for 10 min, and finally, a gradual rise to 290 °C at a rate of 25 °C/min. The transfer line temperature and ion source temperature were set at 310 °C and 300 °C, respectively. Mass spectrometer parameters for the various DDT and DDT metabolites are detailed in Table 3. 

Between each sample, blanks containing only solvent were analyzed to confirm the absence of interference peaks. Calibration standard solutions were assessed both at the beginning and end of each sequence. The degradation of DDT during the analysis was examined before the run and determined to be less than 5%. The calibration curve was constructed by plotting concentrations against the ratio of the analyte peak area to the peak area of the internal 13C-labeled o,p’-DDT standard.

### 2.7. Statistical Analysis 

Analysis of variance (ANOVA) was conducted, with the concentration of either DDT or DDT metabolites serving as the quantitative factor, and the strategy type, encompassing the rate and type of sequestrant material used, as the qualitative factor. Subsequent to the ANOVA tests, Dunnett post hoc tests were performed using R software (version 4.2.2, Vienna, Austria). Significance was established at a threshold of *p* < 0.05. Each modality had four replicates except for the non-amended (or control) treatment group, which had eight replicates.

## 3. Results 

### 3.1. Granulometry Results of Particles Biochar and Activated Carbon Samples

**Figure 1:**
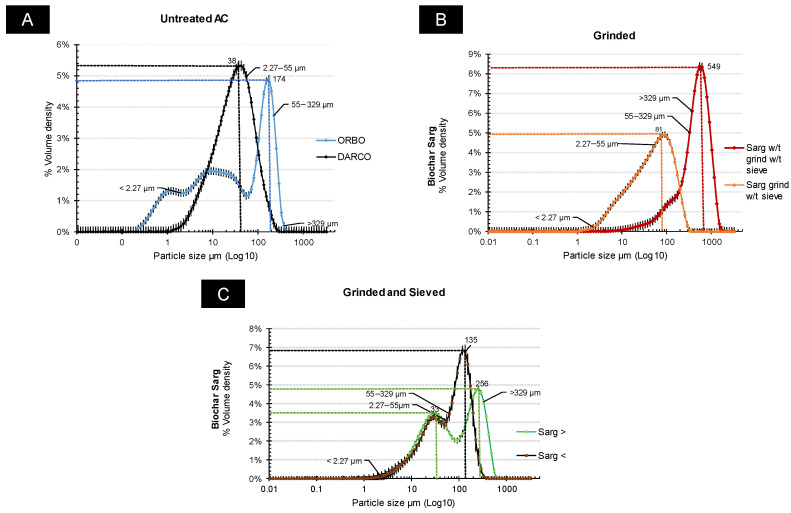
Particle size distribution of (**A**) untreated samples of ACs (ORBO and DARCO), (**B**) ground and mechanically treated samples of BCs Sarg, (**C**) particle size distribution of activated carbon ground and sieved samples of BCs after laser diffraction analysis analyzer (Mastersizer 3000, Malvern Panalytical, Palaiseau, France) (w/t—without) (presented in %Volume density mean ± SE).

An investigation into the granulometry of Sargasso BC and ACs (DARCO and ORBO) enabled their classification into four size classes based on the size range: (1) <2.27 µm; (2) 2.27 to 55 µm; (3) 55 to 329 µm; and (4) >329 µm (Figure 1). 

Within the untreated activated carbon (AC) group, comprising DARCO and ORBO as raw materials, particle distribution in the four classes was predominantly influenced by the second and third classes, ranging from 2.27 to 55 μm and from 55 to 329 μm, respectively (Figure 1A). For DARCO, class 2 contributed significantly to the overall particle distribution, exhibiting a smooth increase in particles (77% of total volume density, with a peak at 174 μm). In contrast, the particle distribution in ORBO displayed a two-fold pattern: firstly, the presence of particles in the finest class (<2.27 μm) accounted for 16% of the total volume; secondly, an equal distribution in the second and third size classes (1:1 ratio) with 42.9% and 40.3% total volume density, respectively. 

Similar size distribution classes were observed for the BC Sarg samples (Figure 1B,C). Pretreatment methods, such as grinding and sieving, influenced the particle size distribution. In the case of raw BC Sarg (untreated), particles from the 4th group (>329 μm) predominated, constituting 62% of the total volume density, with the most intense peak observed at 549 μm. Conversely, in the ground form without sieving, particles from groups 2 and 3 dominated (2: 53% and 3: 45% total volume density) with the most intense peak at 81 μm (Figure 1B).

Within the treatment group involving grinding and sieving for BC Sarg >150 μm, the particle distribution displayed a predominance of class 2 and 3 particle sizes, accounting for total volume densities of 41% and 52%, respectively. The initial peak occurred at 33 μm, with a bulk density of 3.37%, while the second peak was observed at 256 μm, with a bulk density of 4.6%. Classes 1 and 4 of particles were also present, contributing to total bulk densities of 0.80% and 6.5%, respectively (Figure 1C).

For BC Sarg <150 μm, particles were distributed among the 1st, 2nd, and 3rd classes with total volume densities of 1%, 62.7%, and 35.8%, respectively, for each class. Two peaks were observed, with the first at 29 µm, which was followed by an increase to 135 µm (volume density of 3% and 6%). Despite having nearly the same contribution of particle sizes to the total volume in BC samples Sarg >150 μm, an increase in volume density and a prevalence of the 2nd class were observed after grinding and sieving Sarg <150 μm (40.8% and 62.7%). The particle size distribution of the BC Sarg >150 μm sample was observed in a similar order of size classes 2 and 3 as ORBO samples in terms of total volume density (40–52% and 42–40%, respectively). The same similarity was observed for Sarg <150 μm and DARCO samples in terms of total volume density only with a greater predominance of class 2 (class 2–3: 62.7–35.8% and 77–22%, respectively).

### 3.2. Improving a Sargasso Biochar-Based Sequestration Strategy 

**Figure 2:**
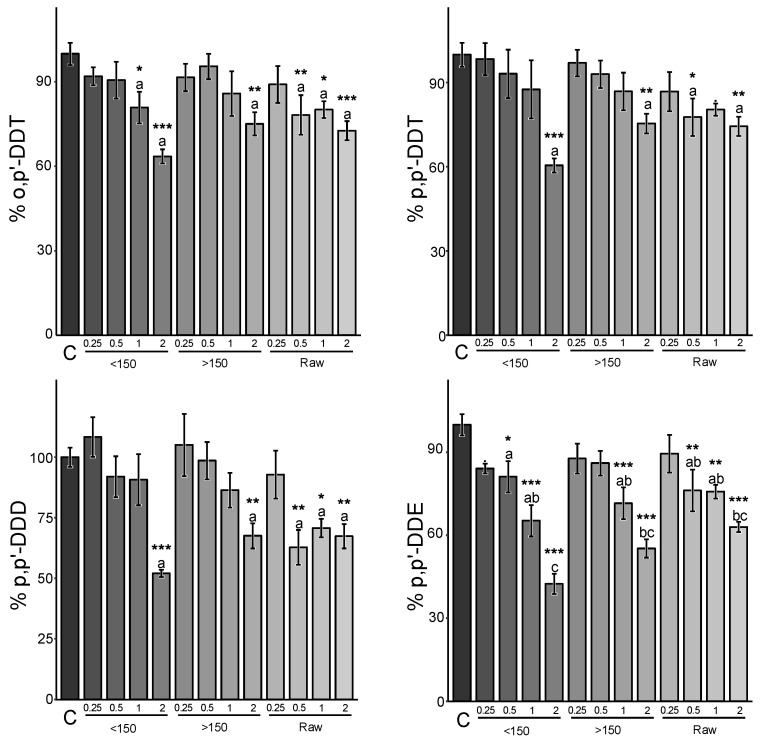
Relative environmental availability of DDTs and DDT metabolites in Kyzylkairat soil following amendment of Sargasso BCs at different rates and particle sizes. The “<150” group represents ground and sieved particles up to <150 µm in the amended groups; “>150” denotes the fraction above 150 µm, and “raw” represents the non-ground, raw BC material. Values from 0.25 to 2 refer to the applied amendment rate. The values correspond to the mean ± standard error (SE) (*n* = 4). Group mean values with superscript asterisks indicate statistical differences from the non-amended soil (C) (0.05: * > 0.01: ** > 0.001: ***) using variance analysis and a Dunnett post hoc test. Groups’ mean concentrations with different superscript letters (a, b, c) are statistically different (*p* < 0.05) from each other using a complementary variance analysis followed by a Tukey post hoc test on previously found significant groups using Dunnett’s.

To enhance the sequestration efficiency of the Sargasso BC amendment on soils contaminated with DDT and DDT metabolites, two factors were evaluated: the amendment rate and the granulometry of the Sargasso BC (Figure 2). 

### 3.3. Amendment Rate Effect 

Only the 2% amendment rate demonstrated a significant difference from the non-amended soil (Figure 2). Starting from this rate, reductions of availability were obtained for p,p’-DDD (37.4 ± 9.1%), p,p’-DDE (46.6 ± 6.7%), o,p’-DDT (29.6 ± 5.9%), and p,p’-DDT (29.9 ± 6.0%) in soil K (mean ± SE). Concerning the BC, the amendment rate emerges as the most significant factor influencing environmental availability: the higher the amount of BC applied, the lower the environmental availability of the different congeners (*p* < 0.001). 

### 3.4. Granulometry Effect 

**Figure 3:**
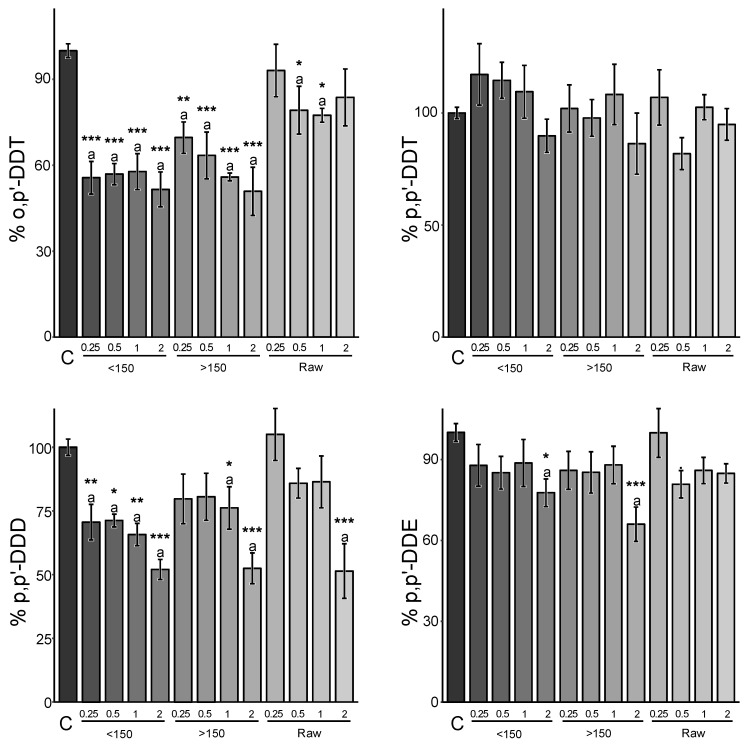
Relative environmental availability of DDTs and DDT metabolites in Beskainar soil following the amendment of Sargasso BCs at different rates and particle sizes. “150” represents the fraction above 150 µm, and “raw” denotes the non-ground, raw BC material. Values from 0.25 to 2 refer to the applied amendment rate. The values correspond to the mean ± standard error (SE) (*n* = 4). Group mean values with superscript asterisks indicate statistical differences from non-amended soil (C) (0.05: * > 0.01: ** > 0.001: ***) using variance analysis and a Dunnett post hoc test. A complementary variance analysis followed by a Tukey post hoc test on previously found significant groups using Dunnett’s shows no statistical differences, presenting the same letter.

A reduction in environmental availability up to 45.6 ± 6.5% (mean of congeners ± SE) was achieved with ground and sieved Sargasso BC (<150 µm), while the highest granulometric fraction exhibited a 31.3 ± 15.5% reduction, similar to the raw material (30.8 ± 9.7%) when amended at 2% rates. Therefore, a potential 1.4 times higher reduction may be observed when using the finest particles compared to the others. Additionally, it is important to note a significant reduction in variability when using the finest fraction. However, these results were not significantly different (*p* > 0.05) for soil B, as more variable results were obtained (Figure 3). 

In light of this preliminary experiment, it becomes evident that an amendment rate of at least 2% is necessary to ensure the effectiveness of the strategy when utilizing Sargasso BC. Additionally, the reduction in particle size seems to contribute to an enhanced reduction. Therefore, the steps of grinding and sieving emerge as crucial measures to implement.

### 3.5. Comparing Sargasso Biochar to Activated Carbon-Based Sequestration Strategies 

The second set of experiments aimed to compare the efficacy of the strategy using Sargasso BC, a cost-effective material, with the more expensive carbonaceous alternatives: high-quality ACs (ORBO, DARCO), introduced at varying rates.

- Carbonaceous matrix effect

**Figure 4:**
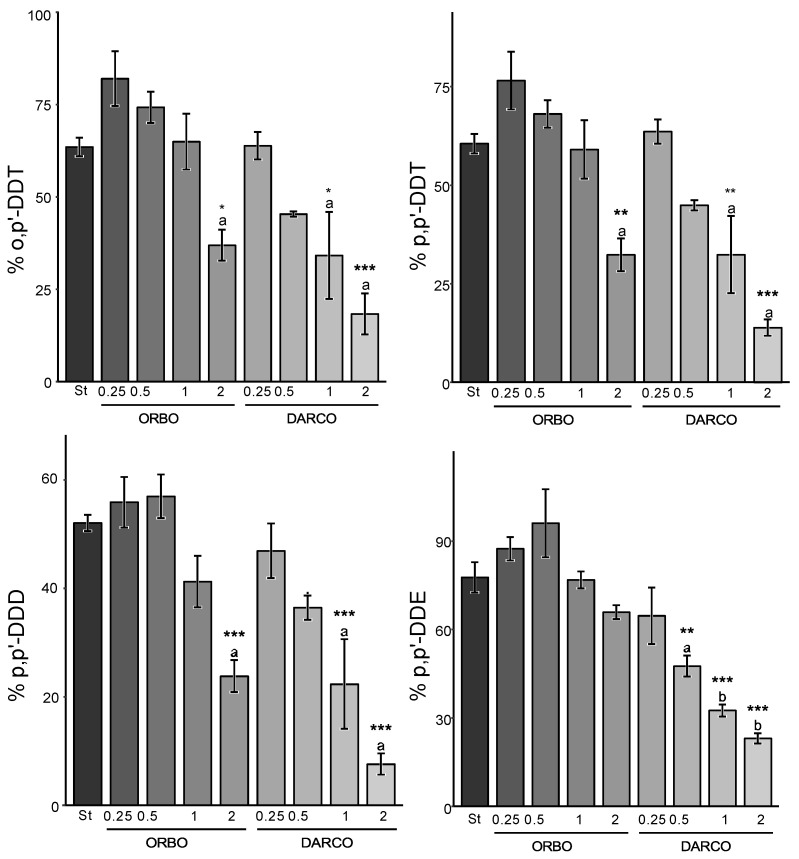
Relative environmental availability of DDTs and DDT metabolites in Kyzylkairat soil, comparing selected BC strategy and AC-based strategy. The “ORBO” group represents amended soil with ORBO, “DARCO” denotes amended soil with DARCO, and “St” represents the selected strategy: amendment of soil by 2% of <150 µm Sargasso BC. Values correspond to the mean ± standard error (SE) (*n* = 4). Group mean values with superscript asterisks indicate statistical differences from the selected strategy (St) (<0.05: * < 0.01: ** < 0.001: ***) using variance analysis and a Dunnett post hoc test. Groups mean concentrations with different superscript letters (a, b) are statistically different (*p* < 0.05) from each other using a complementary variance analysis followed by a Tukey post hoc test on previously found significant groups using Dunnett’s test.

**Figure 5:**
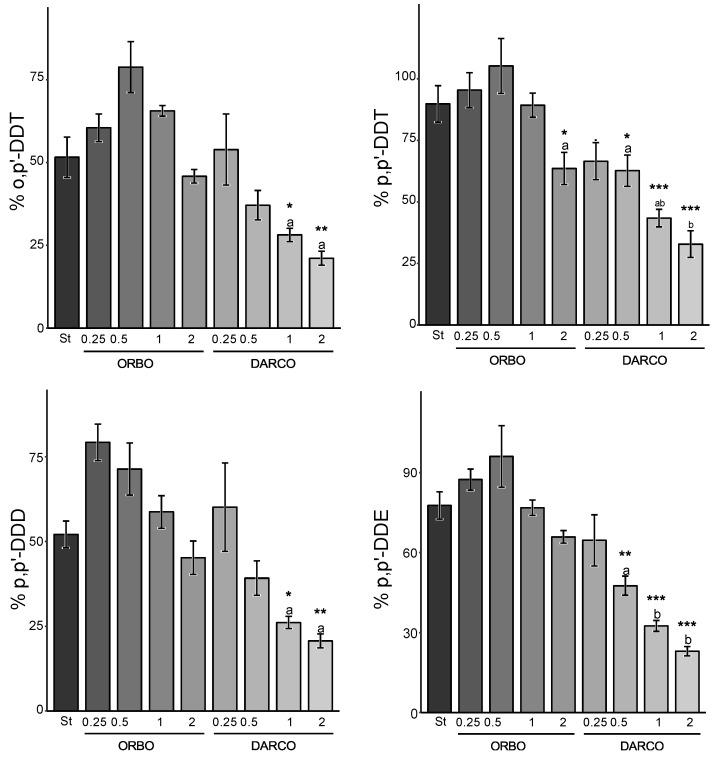
Relative environmental availability of DDTs and DDT metabolites in Beskainar soil, comparing selected BC strategy and AC-based strategy. The “ORBO” group represents amended soil with ORBO, “DARCO” denotes amended soil with DARCO, and “St” represents the selected strategy. Values correspond to the mean ± standard error (SE) (*n* = 4). Group mean values with superscript asterisks indicate statistical differences from the selected strategy (St) (<0.05: * < 0.01: ** < 0.001: ***) using variance analysis and a Dunnett post hoc test. Groups mean concentrations with different superscript letters (a, b) are statistically different (*p* < 0.05) from each other using a complementary variance analysis followed by a Tukey post hoc test on previously found significant groups using Dunnett’s test.

The AC-based sequestration strategy proved to be significantly more effective than the BC-based ones (Figure 4 and Figure 5). On average, the reductions offered by ORBO appear to be less than DARCO for the same congener, soil, and amendment rate (by a mean factor of 1.2). This result contrasts with the specific surface area findings, as ORBO presents the most significant surface area (Table 2). However, when compared to Sargasso BC at the same application rate, BC appears to be the less effective material, even if its surface area is similar to DARCO’s (*p* < 0.001).

- Soil effect

Another notable difference in terms of environmental availability responses was observed when comparing both studied soils. Specifically, the environmental availability (%) of DDT and its metabolites appeared to be more reduced in Kyzylkairat than in Beskainar soil when implementing the same amendment and application rate (Figure 2 and Figure 3). These differences may be attributed to variations in concentrations found in both soils. Before amendment, soil K exhibited the highest environmental availability for each compound (3960 ± 220 µg kg^−1^ of p,p’-DDE; 74.4 ± 4.4 µg kg^−1^ of p,p’-DDD; 1290 ± 70 µg kg^−1^ of o,p’-DDT and 3750 ± 210 µg kg^−1^ of p,p’-DDT) up to two magnitudes higher than the environmental availability of non-amended soil B (18.4 ± 0.7 µg kg^−1^ of p,p’-DDE; 91.5 ± 3.3 µg kg^−1^ of p,p’-DDD; 1.7 ± 0.1 µg kg^−1^ of o,p’-DDT and 4.7 ± 0.2 µg kg^−1^ of p,p’-DDT).

- Congener of DDT and DDT metabolites

Finally, the congeners also displayed varying responses to such sequestration strategies. In soil K, p,p’-DDD appeared to be the most susceptible congener to this sequestration strategy, followed by p,p’-DDE, whereas the parent compounds o,p’-DDT and p,p’-DDT were the most recalcitrant to it. In soil B, the same order was observed, with the notable exception of o,p’-DDT, for which sequestration appeared to be significantly greater than in soil K (Figure 5). These similarities between (i) DDTs on one hand and (ii) DDD and DDE on the other hand were expected, as they possess very similar characteristics and the same chemical formula.

- Rate of amendment effect

Another prominent effect is the amendment rate in the AC-based sequestration strategy. A linear reduction trend in the environmental availability of each congener was observed with respect to the amendment rate of AC for each congener. Taking p,p’-DDT and ORBO amendment as an example, an 18% reduction in o,p’-DDT was observed at a 0.25% amendment rate in Kyzylkairat soil, increasing to 63% when amended by 2%. The linearity of the reduction suggests that doubling the application rate results in an additional 8–13% reduction. For the same congener and soil but with DARCO application, a 0.25% rate resulted in a 36% reduction, while a 2% application led to an 82% reduction. Again, the linear reduction trend indicates that doubling the application rate results in an additional 11–16% reduction.

In comparison to BC, even a 0.5% amendment rate (four times less) of DARCO provides similar to better sequestration than the highest amendment rate of BC, and the value is 1% for ORBO (two times less).

- Expected reduction in environmental availability using such strategies

In order to predict the impact of such a strategy in terms of reduction in transfer, a 90th confidence interval was calculated from all the tested modalities presented above (Table 4 and Table 5). The obtained result demonstrated a possible high reduction in transfer of DDT and DDT metabolites with a minimum of 80% reduction for a 2% application rate of DARCO in Kyzylkairat (p90; p,p’-DDT) and a minimum of 57% reduction in Beskainar (p90, p,p’-DDT). Alternatively, a 2% application rate of Sargassum BC ground and sieved until 150 µm showed a minimal reduction of 31% (p90 o,p’-DDT) in Kyzylkairat. However, such a strategy was found to be non-efficient for the less contaminated soil Beskainar, as a reduction of 0% was calculated as p90 for p,p’-DDT.

## 4. Discussion 

### 4.1. Particle Size and Amendment Rate Impact on DDT and DDT Metabolites Sequestration 

The outcomes of laser diffraction analysis on particles of untreated AC and treated BC revealed that mechanical processing significantly impacts their particle size distribution, leading to an enhancement in sequestration efficiency and a marked reduction in the environmental availability of DDT and DDT metabolites in soils K and B. Post-treatment processes, such as grinding and sieving, are known to influence the physical characteristics of BC [29,30,31], affecting its potential applications, including water retention [29] and the sequestration of soil-bound persistent organic pollutants (POPs) like chlorinated pesticides [32].

This effect may arise from improved contact between the contaminated soil particles and the BC when the carbonaceous particle size is smaller [33,34,35]. Another possibility is that the porosity of BC may be enhanced through grinding and sieving. While published data indicate a negative correlation between particle size and specific surface area (SSA) [24], the present study observed a slight decrease in SSA for Sargassum BC after grinding and sieving. This finding contrasts with recent research on another POP (chlorinated pesticides), where the sequestration efficiency of carbonaceous materials was positively correlated with their specific surface area [33,34,35]. A larger surface area implies more sites for potential adsorption of POPs, explaining this positive correlation.

An alternative explanation for the observed improvements post-processing is a better interaction and greater contact surface due to the small size of BC particles with those of the soil, facilitating the transfer of pollutants into the porous network of the carbonaceous material. This explanation may also be applicable to the observed amendment rate effect. As anticipated, an increase in BC amendment rate in soils K and B resulted in a decrease in environmentally available concentrations of DDT and DDT metabolites. These findings align with previous research highlighting the significance of the amendment rate in the sequestration of DDTs by ACs [36] and other POPs [32]. The logical consequence of this increase is an elevation in contact surfaces with contaminated soil particles, leading to the enhanced trapping of DDTs and their metabolites. 

These considerations may also elucidate the varying effectiveness between the chosen strategy (2% amendment rate, <150 µm) and those involving AC DARCO or ORBO. Despite the two ACs having higher specific surface areas (SSA) than BC, they are generally more effective than the selected strategy at the same amendment rate. However, ORBO, with the highest SSA, appears to be as effective as Sargasso BC <150 µm for the less contaminated B soil and less efficient than the selected strategy before 1% for the highly contaminated K soil. This could be attributed to the distinctive microporosity of ORBO, whereas DARCO and Sargasso BCs are recognized for their mesoporosity [25]. Furthermore, DARCO not only boasts the highest SSA but also the finest particle size among all studied materials, elucidating its efficacy in limiting the environmental availability of DDT and DDT metabolites compared to others.

### 4.2. Implement Such a Strategy on Contaminated Areas 

The results of both experiments present promising prospects for soils with varying contamination levels. They underscore the efficacy of carbon-rich materials derived from Sargassum in reducing the environmental availability of the studied compounds. However, it is important to note that the current experiments solely address the sequestration aspect of BC and AC materials, without investigating their potential impact on soil functionality, particularly with regard to safe food production. Future studies may delve into these broader aspects to provide a more comprehensive understanding of the materials’ effects on soil health and productivity.

BCs/ACs are generic adsorbents that may, on the one hand, modulate the availability of chemical elements in the soil and, thus, their uptake by plants. Ultimately, such an addition may impact soil fertility and crop production. In this perspective, the literature provides contrasted results highlighting the positive or negative impacts of BCs on crop yields. As an illustration, Kim et al. 2015 observed that 0.5 and 1% amendment rates of orange pulp-based BC caused an increase in lettuce biomass, while 2%, 5% and 10% rates led to a reduction [37]. It is crucial to note that the amendment rate of BC in the soil could be a critical factor influencing its impact on DDT sequestration, as shown in the present results. Such observations are in line with previous results.

On the other hand, it is important to recognize that the raw materials used in BC/AC production may exert detrimental effects on soil functions. For example, BC could contain consistent levels of trace metals, particularly where they originate from manure. Additionally, BC derived from *Sargassum* spp. is known to exhibit variable concentrations of heavy metals, notably arsenic. Consequently, such BC may release this toxic element into the soil, potentially jeopardizing its fertility. Therefore, in forthcoming studies, it is imperative to consider the impact of BCs/ACs on soil ecosystems when determining the appropriate amendment rate. Essentially, a delicate balance must be struck between sequestration efficiency and potential repercussions on soil fertility. 

Due to the relatively recent investigations onto the use of BCs for remediation purposes [38], studies on BCBC behavior have focused more on short-term treatments than in long-term options. The long-term impacts of BCs on soil remain poorly understood [39], which was primarily because highly pyrolyzed BC has a minimum estimated half-life of 1000 years [40], raising concerns about potential effects on the soil environment. External factors such as temperature changes (freezing–thawing periods) [41], dry–moist cycles, solar radiation, and reduction in oxidation lead to significant changes in the physicochemical properties (specific surface area, chemical surface groups, pH, etc.) of BC that can cause various effects in the soil (positive, negative, or no effect) [42,43]. The impact of BC treatment may affect soil aggregation [44]. In this frame, further investigations about the innocuity of such application are still needed and appear to be highly dependent on the characteristics of the studied BC.

## 5. Conclusions

A sequestration strategy involving highly carbonaceous materials has demonstrated a significant reduction in the relative environmental availability of DDT and its metabolites, making it a compelling option for the remediation of contaminated soils. This strategy proved particularly effective for BC after undergoing grinding and sieving processes to reduce particle size, especially when applied at a minimum rate of 2% amendment. In such cases, reduction levels exceeding 40% were observed for all studied compounds. The AC-based sequestration strategy appeared even more effective, achieving higher reduction levels (up to 90%) for soils amended at 2%. In both strategies, increasing the amendment rate resulted in improved efficiency. Additionally, a notable congener effect was observed with sequestration efficiency following the order: p,p’-DDE = p,p’-DDD > p,p’-DDT = o,p’-DDT. Given the cost-effectiveness of BCs compared to ACs, which involve more expensive activation processes during production, further investigation is warranted to (i) optimize the sequestration potential pre-application and (ii) assess the safety of such amendments.

## Figures and Tables

**Table 1:** Physical characterization of the different carbonaceous matrix.

	Feedstock	Pyrolysis	Activation	Grinding–Sieving	Textural Parameters	
					BET Surface Area (m^2^·g^−1^)	V_Micropores_ (D-R) (cm^3^·g^−1^)
Sarg	*Sargassum* spp.	700 °C for 3 h	None		582	0.15
Sarg<	*Sargassum* spp.	700 °C for 3 h		2 min grinding and sieved < 150 µm	497.6	0.12
Sarg>	*Sargassum* spp.	700 °C for 3 h		2 min grinding fraction > 150 µm	567.0	0.15
ORBO^TM^	Coconut	N/A	N/A		1127	0.34
DARCO^©^	Lignite	N/A	Physical (water)		793.8	0.17

N/A: information is not provided by the manufacturer. BET Surface area—Brunauer–Emmett–Teller (BET) surface area. V_Micropores_—microporous volume.

**Table 2:** Soil DDT and DDT metabolite concentrations (µg/kg of dry matter).

	Kyzylkairat	Beskainar
DDD-4,4′ (p,p’-DDD)	852	8.47
DDE-4,4′ (p,p’-DDE)	6035	276
DDT-2,4′ (o,p’-DDT)	906	48.3
DDT-4,4′ (p,p’-DDT)	2277	10.6

**Table 3:** Mass spectrometer parameters of DDT and DDT metabolites analysis.

Congener	Retention Time (min)	Mass	Product Mass	Collision Energy (eV)	Quantification (Q) or Qualification (-)
p,p’-DDE	27.01	246	176.1	28	Q
	27.01	317.8	246	20	
	27.01	317.8	248	18	
o,p’-DDT	29.66	235	165.1	22	Q
	29.66	235	199.5	10	
	29.66	236.8	165	22	
p,p’-DDD	29.73	235	165.1	20	Q
	29.73	235	199	14	
	29.73	236.8	165	20	
p,p’-DDT	30.45	235	165.1	22	Q
	30.45	235	199.5	10	
	30.45	236.8	165	22	
^13^C o,p’-DDT	29.66	247	177.1	22	Q
	29.66	177.1	176.1	22	
	29.66	249	177.1	20	

**Table 4:** Environmental availability means, 90th confidence intervals and reduction factors of Kyzylkairat soil. The 90th confidence intervals were derived from relative environmental availability data and reduction factors upon P-90. Values beneath 0 were replaced by “-“.

Amendment Material		Amendment Rate	90th Confidence Interval								Reduction Factor			
			p,p’-DDD		p,p’-DDE		o,p’-DDT		p,p’-DDT		p,p’-DDD	p,p’-DDE	o,p’-DDT	p,p’-DDT
Sargasso biochar	<150	0.25%	108%	[89, 128]	84%	[80, 88]	92%	[84, 100]	98%	[85, 112]	-	12%	-	-
		0.5%	92%	[72, 112]	81%	[68, 95]	91%	[75, 106]	93%	[73, 113]	-	5%	-	-
		1%	91%	[66, 116]	65%	[52, 79]	81%	[68, 94]	88%	[63, 112]	-	21%	6%	-
		2%	52%	[49, 56]	42%	[34, 51]	64%	[58, 69]	61%	[55, 66]	44%	49%	31%	34%
	>150	0.25%	105%	[75, 135]	88%	[75, 101]	92%	[80, 103]	97%	[86, 108]	-	-	-	-
		0.5%	99%	[80, 117]	86%	[76, 97]	96%	[85, 106]	93%	[82, 105]	-	3%	-	-
		1%	86%	[69, 103]	72%	[58, 85]	86%	[67, 105]	87%	[71, 103]	-	15%	-	-
		2%	68%	[55, 80]	55%	[47, 63]	75%	[65, 85]	75%	[67, 84]	20%	37%	15%	16%
	All	0.25%	93%	[69, 116]	89%	[73, 106]	89%	[74, 105]	87%	[70, 103]	-	-	-	-
		0.5%	63%	[46, 80]	76%	[58, 94]	78%	[62, 95]	78%	[62, 93]	20%	6%	5%	7%
		1%	71%	[62, 80]	76%	[70, 82]	80%	[73, 87]	80%	[75, 86]	20%	18%	13%	14%
		2%	67%	[56, 79]	63%	[59, 67]	73%	[65, 81]	74%	[66, 83]	21%	33%	19%	17%
ORBO		0.25%	56%	[45, 67]	66%	[49, 84]	82%	[65, 99]	77%	[59, 94]	33%	16%	1%	6%
		0.5%	57%	[47, 66]	51%	[41, 62]	74%	[64, 84]	68%	[60, 76]	34%	38%	16%	24%
		1%	41%	[30, 52]	36%	[19, 53]	65%	[47, 83]	59%	[42, 76]	48%	47%	17%	24%
		2%	24%	[17, 31]	19%	[13, 25]	37%	[27, 47]	32%	[22, 42]	69%	75%	53%	58%
DARCO		0.25%	47%	[35, 59]	56%	[46, 66]	64%	[55, 73]	64%	[56, 71]	41%	34%	27%	29%
		0.5%	36%	[31, 42]	38%	[36, 39]	45%	[44, 47]	45%	[42, 48]	58%	61%	53%	52%
		1%	22%	[3.0, 42]	23%	[10, 35]	34%	[6.5, 62]	32%	[9.3, 55]	58%	65%	38%	45%
		2%	7.6%	[3.6, 12]	15%	[5.2, 24]	18%	[5.3, 31]	14%	[9.6, 18]	88%	76%	69%	82%

**Table 5:** Environmental availability means and 90th confidence intervals and reduction factors of Beskainar soil. The 90th confidence intervals were derived from relative environmental availability data and reduction factors upon P-90. Values beneath 0 were replaced by “-“.

Amendment Material		Amendment Rate	90th Confidence Interval								Reduction Factor			
			p,p’-DDD		p,p’-DDE		o,p’-DDT		p,p’-DDT		p,p’-DDD	p,p’-DDE	o,p’-DDT	p,p’-DDT
Sargasso biochar	<150	0.25%	71%	[54, 87]	88%	[70, 106]	56%	[42, 69]	117%	[85, 149]	13%	-	31%	-
		0.5%	71%	[65, 77]	85%	[71, 99]	57%	[48, 66]	115%	[91, 138]	23%	1%	34%	-
		1%	66%	[55, 76]	89%	[68, 109]	58%	[43, 73]	109%	[82, 137]	24%	-	27%	-
		2%	52%	[43, 61]	78%	[66, 90]	52%	[34, 69]	90%	[72, 107]	39%	10%	31%	-
	>150	0.25%	80%	[57, 103]	86%	[69, 103]	70%	[57, 83]	102%	[77, 127]	-	-	17%	-
		0.5%	81%	[59, 102]	85%	[67, 103]	63%	[44, 83]	98%	[79, 117]	-	-	17%	-
		1%	76%	[57, 96]	88%	[72, 104]	56%	[53, 59]	108%	[77, 140]	4%	-	41%	-
		2%	53%	[38, 67]	66%	[51, 81]	51%	[31, 71]	86%	[54, 118]	33%	19%	29%	-
	All	0.25%	105%	[81, 129]	100%	[79, 121]	93%	[72, 115]	107%	[78, 136]	-	-	-	-
		0.5%	86%	[72, 100]	81%	[69, 93]	79%	[60, 99]	82%	[65, 99]	0%	7%	1%	1%
		1%	86%	[63, 110]	86%	[74, 97]	77%	[72, 83]	103%	[89, 116]	-	3%	17%	-
		2%	51%	[20, 83]	85%	[76, 93]	84%	[21, 146]	95%	[78, 112]	17%	7%	-	-
ORBO		0.25%	79%	[67, 92]	87%	[78, 97]	60%	[51, 70]	95%	[79, 112]	8%	3%	30%	-
		0.5%	71%	[49, 94]	96%	[69, 123]	79%	[61, 97]	105%	[79, 132]	6%	-	3%	-
		1%	59%	[45, 73]	77%	[68, 85]	66%	[56, 76]	89%	[75, 104]	27%	15%	24%	-
		2%	45%	[34, 57]	66%	[60, 71]	46%	[41, 51]	64%	[48, 79]	43%	29%	49%	21%
DARCO		0.25%	60%	[29, 91]	65%	[42, 87]	54%	[29, 79]	67%	[49, 84]	9%	13%	21%	16%
		0.5%	39%	[27, 51]	48%	[39, 56]	37%	[27, 48]	63%	[48, 78]	49%	44%	52%	22%
		1%	26%	[22, 30]	33%	[28, 37]	28%	[23, 33]	43%	[35, 52]	70%	63%	67%	48%
		2%	21%	[15, 27]	23%	[18, 28]	21%	[15, 27]	33%	[17, 49]	73%	72%	73%	51%

## Data Availability

The data presented in this study are openly available in: Delannoy, Matthieu, 2024, “The Effect of Granulometry of Carbonaceous Materials and Application Rates on the Availability of Soil-Bound Dichloro-diphenyltrichloroethane (DDT) and Its Metabolites”, https://doi.org/10.57745/NWZOAE, Recherche Data Gouv (https://entrepot.recherche.data.gouv.fr/).
